# Lack of *T04C9.1*, the Homologue of Mammalian APPL2, Leads to Premature Ageing and Shortens Lifespan in *Caenorhabditis elegans*

**DOI:** 10.3390/genes15060659

**Published:** 2024-05-22

**Authors:** Zirui Li, Zhiqiang Chen, Lianghao Zhao, Jiaqi Sun, Lin Yin, Yuwei Jiang, Xiaotong Shi, Ziye Song, Lu Zhang

**Affiliations:** College of Bioengineering, Henan University of Technology, 100 Lianhua Street, High-Tech Zone, Zhengzhou 450001, China

**Keywords:** *C. elegans*, *T04C9.1*, APPL2, ageing, autophagy, lifespan

## Abstract

Ageing has been identified as an independent risk factor for various diseases; however, the physiological basis and molecular changes related to ageing are still largely unknown. Here, we show that the level of APPL2, an adaptor protein, is significantly reduced in the major organs of aged mice. Knocking down APPL2 causes premature ageing of human umbilical vein endothelial cells (HUVECs). We find that a lack of *T04C9.1*, the homologue of mammalian APPL2, leads to premature ageing, slow movements, lipid deposition, decreased resistance to stresses, and shortened lifespan in *Caenorhabditis elegans* (*C. elegans*), which are associated with decreased autophagy. Activating autophagy by rapamycin or inhibition of *let-363* suppresses the age-related alternations, impaired motility, and shortened lifespan of *C. elegans*, which are reversed by knocking down autophagy-related genes. Our work provides evidence that APPL2 and its *C. elegans* homologue *T04C9.1* decrease with age and reveals that a lack of *T04C9.1* bridges autophagy decline and ageing in *C. elegans*.

## 1. Introduction

Population ageing has become a major concern worldwide; it is estimated that by 2030, people aged ≥ 60 years will account for 20% of the world’s total population, with an exponential increase in all age-related diseases [1]. The ageing process leads to a gradual decline in normal tissue functions, which worsens body functions and eventually leads to a variety of age-related diseases, including metabolic and musculoskeletal disorders, cardiovascular diseases, neurodegenerative diseases, and cancer [2,3]. Although numerous diseases related to ageing and premature ageing have been pathologically identified in humans, the physiological basis and underlying molecular mechanisms of ageing are still largely unknown. Therefore, identifying the molecular changes linked with ageing is highly desirable for developing interventions to delay ageing and the goal of extending lifespan.

The APPL (adaptor protein containing pleckstrin homology domain, phosphotyrosine domain, and leucine zipper motif) family consists of two proteins, APPL1 and APPL2, which are a pair of endosomal and signalling molecules with the same domain organisation and 54% identity in protein sequences [4]. APPL1 is the first-identified member with the function of interacting with adiponectin receptors and adiponectin signalling, as well as with metabolically regulated insulin signalling pathways [5]. Subsequently, APPL1 was widely studied and found to be related to multiple physiological functions such as cell migration, energy metabolism, oxidative stress, and inflammation regulation [6]. APPL1 is down-regulated in the plasma and myocardium of aged mice and when APPL1 is knocked down, adiponectin’s ability to resist D-galactose-induced myocardial ageing is lost [7]. Unlike its homologous APPL1, although the essential role of APPL2 in cell survival, proliferation, cytokine, and insulin secretion was uncovered in recent years [8,9,10], its physiological function, especially whether this adaptor protein changes with age and its role in ageing, has never been explored.

The short lifespan of *Caenorhabditis elegans* (*C. elegans*), its genetic tractability, and its apparent age-dependent physiological alternations, as well as being free of ethical concerns in *C. elegans* experiments, make this species a favourite model organism for ageing research [11]. The use of *C. elegans* has established the causal links between numerous conserved molecular signalling pathways and the ageing process, including insulin/insulin-like growth factor (IGF), hypoxia-inducible factor 1, target of rapamycin (TOR), mitochondrial function pathways, and so on [12]. More recent work using this model led to the discovery of novel molecular targets that regulate ageing, such as KLF family transcription factors and CD44 [13,14]. In this study, we find that the levels of APPL2 in the major organs of aged mice were significantly lower than those of young mice and knocking down APPL2 induces premature senescence of human umbilical vein endothelial cells (HUVECs). Therefore, we decided to investigate whether *T04C9.1*, an APPL2 homologue, regulates ageing and lifespan in *C. elegans*. Here, we demonstrated that the lack of *T04C9.1* promotes ageing and shortens lifespan in *C. elegans* by inhibiting autophagy. Investigation of *T04C9.1* deficiency-mediated shortened lifespan in *C. elegans* will contribute to a better understanding of the molecular changes in the ageing process.

## 2. Materials and Methods

### 2.1. Materials

The details of the antibodies, reagents, cells, animals, plasmids, and software used in this study are listed in Supplementary Table S1.

### 2.2. Animals and Treatments

Six-week-old C57BL/6 mice (purchased from The Laboratory Animal Center of Zhengzhou University, Zhengzhou, China) were housed in a temperature and humidity-controlled room (25 °C and 40–60%, respectively) on a 12 h light/dark cycle from 8 a.m. to 8 p.m., with free access to water and chow diet. We used 3-month-old mice as young mice and 21- to 26-month-old mice as aged mice. The mice were euthanised and different organs and aorta were collected for subsequent research. All experiments were carried out following the Guide for the Care and Use of Laboratory Animals published by the US National Institutes of Health (NIH Publication No. 85–23, revised 1996) and approved by the Animal Care and Use Committee of Zhengzhou University (Approval no. ZZU-LAC20231211 [01]).

Nematodes were cultured and propagated on nematode growth media (NGM) agar plates with *Escherichia coli* (*E. coli*) strain OP50 at 20 °C according to standard techniques. RNAi experiments were conducted by feeding nematodes with HT115 (DE3) bacteria transformed with an L4440-derived plasmid that expresses double-stranded (ds) RNA, as described previously [15]. The plasmids expressing dsRNA against *T04C9.1*, *let-363*, *unc-51*, *vps34*, *atg-7*, and *atg-2* were made by cloning PCR products amplified from cDNA into the vector L4440. The *T04C9.1* fragment was obtained with the following rimers: *T04C9.1* forwards 1, ACACGATCTTTCGGAGTCGG; reverse 1, CGATGATGAGTCCAACGTGC; forwards 2, ACACGATCTTTCGGAGTCGG; reverse 2, TAGTTTTGCCGTGTCAGCGA; forwards 3, CTGCCGGAGAGAATCCATCG; reverse 3, CCGACTCCGAAAGATCGTGT; *let-363* fragment with GCAGCCACAACTTCCAATCG and GGACAAGCCATTCAACACCT; *unc-51* fragment with GTGCTCTCCGAATCTACGGG and GGTATGCACTTGGACCTGCT; *vps34* fragment with AGAAGACTCGTTGCACAATCC and CAGGCCGAAACAATCCCAAC; *atg-7* fragment with AAAGCCGAAACAGCACAAGC and ATCTTCCCAGAAAACCGCGA; and *atg-2* fragment with TTGATGACGGACCCAACCAG and CTTCGTCACCCATTCCGTCA. For rapamycin treatment, 50 mg/mL rapamycin was added to the NGM agar plates at a concentration of 100 μM. For CQ treatment, CQ was added to the NGM agar plate at a concentration of 100 mM for 18 h.

### 2.3. Cell Culture, Transfection, and SA-β-Gal Staining

HUVECs were isolated from the umbilical veins of the umbilical cords of healthy pregnant women using collagenase digestion (0.25 mg/mL) in our lab. The umbilical cords are regarded as medical waste, do not require any additional invasive collection procedures, and do not bear the ethical issues that plague other sources of human vascular endothelial cells. HUVECs were cultured on gelatin-coated plastic dishes in Endothelial Cell Medium (ECM) containing 5% foetal bovine serum (FBS), endothelial cell growth supplement (1×), and 100 U/mL penicillin-streptomycin in a humidified incubator at 37 °C with 5% CO_2_. HUVECs were identified by the typical phase contrast “cobblestone” morphology and the strong positive immunoreactivity to CD31. The cells were subjected to serial passaging to induce senescence. Lentivirus-mediated small hairpin RNA (shRNA)-mediated knockdown of APPL2 was performed as described previously [14]. The oligonucleotide for targeting APPL2 was synthesized and inserted into the pLKO.1-TRC cloning vector. The shRNA sequences targeting APPL2 were as follows: shAPPL2 forwards 1, CCGGGCAAGCAGTGACTCCCATTACCTCGAGGTAATGGGAGTCACTGCTTGCTTTTTG; reverse 1, AATTCAAAAAGCAAGCAGTGACTCCCATTACCTCGAGGTAATGGGAGTCACTGCTTGC; forwards 2, CCGGGCCTGGAACGCCGATTCAATTCTCGAGAATTGAATCGGCGTTCCAGGCTTTTTG; reverse 2, AATTCAAAAAGCCTGGAACGCCGATTCAATTCTCGAGAATTGAATCGGCGTTCCAGGC; forwards 3, CCGGGCTGCTCGGGCTATTCATAACCTCGAGGTTATGAATAGCCCGAGCAGCTTTTTG; and reverse 3, AATTCAAAAAGCTGCTCGGGCTATTCATAACCTCGAGGTTATGAATAGCCCGAGCAGC. HEK293T cells were co-transfected with 1 µg shAPPL2 or pLKO.1 plasmid with 250 ng PMD2.G and 750 ng PsPAX2 using Lipo2000 Transfection Reagent. After 48 h, the medium containing lentivirus was collected and centrifuged at 6000 rpm for 30 min. The determination of SA-β-gal activity was performed through the X-gal staining method using the Senescence β-Gal Staining Kit according to the manufacturer’s protocol. The percentage of blue-stained HUVECs was calculated under a microscope (DM IL LED Fluo, Leica Microsystems CMS GmbH, Wetzlar, Germany). 

### 2.4. RNA Extraction and qRT-PCR

Total RNA was purified from mouse tissues using the RaPure Total RNA Kit (Magen, Guangzhou, China)and extracted from *C. elegans* using the NGzol RNA Uptake Kit (HLingene, Shanghai, China), respectively, according to the manufacturer’s instructions. qRT-PCR was performed as described previously [14]. The primers used for qRT-PCR are listed in Supplementary Table S2.

### 2.5. Protein Extraction and Western Blot Analysis

Mouse organ tissues (20 mg per sample) were lysed and homogenized for 1 min at 4 °C in a Bullet Blender Storm (Next Advance Inc., Averill Park, NY, USA) at speed 3. A total of 2000 nematodes per sample were lysed and total protein was extracted using an ultrasonic cell homogenizer (Ningbo Scientz Biotechnology, Ningbo, China). The processing parameters were ultrasonic power 200 W, work/interval time 10 s/10 s, and total working time 50 min. All homogenates were centrifuged at 12,000 rpm at 4 °C for 10 min and the supernatants were collected for Western blot analysis. Western blot analysis was performed as described previously [14].

### 2.6. Immunohistochemistry (IHC) Staining

The mouse aortic arches were fixed with 4% paraformaldehyde, dehydrated, embedded in paraffin, and sliced into 4-μm sections with an ultrathin semiautomatic microtome (RM2016, Leica, Wetzlar, Germany) according to standard protocols. The slides were uniformly covered with 3% BSA in the chemical circle and closed for 30 min at room temperature; then, they were incubated with the primary antibody against APPL2 (1:200) overnight at 4 °C. The next day, the slides were incubated with goat anti-rabbit HRP secondary antibody (1:200) for 30 min followed by 3,3′-diaminobenzidine (DAB) and hematoxylin staining, respectively. The slides were photographed using a Pannoramic 250 FLASH Pathology Diagnostic System (3DHISTECH, Budapest, Hungary). The DAB staining was analysed by Image-Pro Plus 6.0 software.

### 2.7. C. elegans Lifespan and Reproduction Analysis

The synchronized eggs obtained by bleaching adult nematodes were added to NGM agar plates seeded with *E. coli* strain OP50 at 20 °C. L1 larvae were transferred daily to NGM agar plates containing HT115 bacteria expressing an empty vector L4440 or *T04C9.1* dsRNA, *let-363* dsRNA, *unc-51* dsRNA, *vps34* dsRNA, *atg-7* dsRNA or *atg-2* dsRNAor rapamycin to avoid confusing the original nematodes with their progeny until they stopped laying eggs. The nematodes were then transferred to new NGM agar plates every 2–3 days for the rest of the lifespan experiment. The viability of nematodes was scored every day. Death was defined by a lack of response to a gentle touch with a platinum wire. Nematodes were censored if they escaped, suffered from internal hatching, ruptured, or crawled off the plates. Each lifespan assay was performed using more than 100 animals per group, while the valid number of animals was 100–120. For *C. elegans* reproduction analysis, a total of 30 age-synchronized L4 larvae were transferred to NGM agar plates seeded with either HT115 bacteria expressing an empty vector L4440 or *T04C9.1* dsRNA. The nematodes were transferred to fresh NGM dishes daily until the end of the reproductive period. Eggs were hatched and counted at stage L2 or L3. The daily and total fecundity of each worm were recorded.

### 2.8. Lipofuscin and Lipid Accumulation Assay

The synchronized eggs were added to NGM agar plates seeded with *E. coli* strain OP50 at 20 °C. L1 larvae were transferred to NGM agar plates containing HT115 bacteria expressing an empty vector L4440 or target gene dsRNA daily. On day 7 of adulthood, a total of 20–30 nematodes from each group were placed onto 2% agarose pads coated with 5 mM sodium azide. Images of lipofuscin autofluorescence were obtained using a fluorescence microscope (Axio Observer 3, Carl Zeiss, Oberkochen, Germany). The autofluorescence of lipofuscin in *C. elegans* was quantified as an ageing index. For measurement of lipid accumulation, the Oil Red O staining was performed as previously described [16,17]. On day 7 of adulthood, a total of 20–30 nematodes from each group were collected and washed with M9 buffer containing 0.05% Triton X-100, followed by fixation in 60% isopropanol at room temperature for 3 min. The samples were centrifuged at 560× *g* for 30 s and after the supernatant was discarded, the filtered ORO working solution was added to each sample and incubated at 4 °C overnight. The stained nematodes were cleaned with washing solution and photographed under a microscope (Model Eclipse E200MV RS, Nikon Corporation, Tokyo, Japan).

### 2.9. Pharyngeal Pumping and Body Bending Frequency

The synchronized eggs were added to NGM agar plates seeded with *E. coli* strain OP50 at 20 °C. L1 larvae were transferred to NGM agar plates containing HT115 bacteria expressing an empty vector L4440 or target gene dsRNA or rapamycin daily. On day 7 of adulthood, the plates were placed under a stereomicroscope and magnified enough to clearly observe the pharyngeal pumping and body bending of nematodes. The number of body bends, i.e., the changes in direction of the bend at the mid-body and the number of pumps, i.e., the backward grinder movements in the terminal bulb, were counted every 30 s. A total of 24–40 nematodes from each group were used for each test. Each nematode was videotaped for about 30 s with a CCD video camera under a stereomicroscope (M205, Leica, Wetzlar, Germany).

### 2.10. Stress Resistance Assay

The synchronized eggs were added to NGM agar plates seeded with *E. coli* strain OP50 at 20 °C. L1 larvae were transferred to NGM agar plates containing HT115 bacteria expressing an empty vector L4440 or *T04C9.1* dsRNA daily. On day 7 of adulthood, animals were transferred to NGM agar plates containing 6 mM tBOOH (oxidative stress) or shifted to 35 °C (heat stress). The number of dead nematodes was recorded every 2 h until all the nematodes were dead.

### 2.11. ROS Measurement

The synchronized eggs were added to NGM agar plates seeded with *E. coli* strain OP50 at 20 °C. L1 larvae were transferred to NGM agar plates containing HT115 bacteria expressing an empty vector L4440 or *T04C9.1* dsRNA daily. On day 7 of adulthood, a total of 80 animals in each group were collected and washed with M9 buffer, followed by centrifugation at 560× *g* for 30 s and, after the supernatant was discarded, 50 μM DCFH-DA was added and incubated for 30 min in the dark. Rosup and DCFH-DA were mixed as positive controls. The ROS levels were measured using a fluorescence microplate reader (TECAN, Spark, Männedorf, Switzerland) at excitation and emission wavelengths of 488 nm and 525 nm, respectively, and the values were measured every 20 min for 3 h.

### 2.12. Structure Modelling and Molecular Docking

In order to study the binding mode between the APPL2 and PtdIns3K complex, we constructed the APPL2 structure using Alphafold2 [18,19]. The stereo-chemistry of the best model was analysed by using SAVES V6.0 (https://saves.mbi.ucla.edu/ (accessed on 30 January 2024)). The model was manually checked with WinCoot V0.8.9 [20]. For the PtdIns3K complex structure, by comparison, we used the structure of the latest resolved PtdIns3K complex (PDB ID: 8SOR). The full-length protein binding modes of APPL2 and PtdIns3K complex were studied by using GRAMM-X, which is guided by the experimental details available for their interactions and functions. The protein–protein binding modes were studied and analysed by using the PDBePISA server. The results were analysed by using PyMOL 1.8.6 to characterize critical amino acids in the protein–protein/peptide interaction interface. 

### 2.13. Protein–Protein Interaction Network

The STRING database (https://string-db.org/ (accessed on 14 May 2024)) was applied to construct the protein–protein interactions (PPIs) network. In total, 222 autophagy-related proteins are from the Human Autophagy Database (http://www.autophagy.lu/index.html (accessed on 14 May 2024)). The results were obtained in the STRING online database.

### 2.14. Data Analysis and Statistics

Data analyses were performed by unpaired two-tailed Student’s *t*-test and one-way or two-way ANOVA for multiple groups coupled with Dunnett’s multiple comparisons test using GraphPad Prism 9 for Mac (Version 9.0.0). The log-rank Mantel–Cox test was used to compare the survival curves. All bar graphs show the mean values with error bars (±s.d.). Significance was considered if the *p* value was less than 0.05. The number of repeated experiments and the number of animals are indicated in the figure legends. The bands of the Western blot were quantified using Image Studio Digits software (Version 5.2.5, LI-COR Biotechnology, Lincoln, NE, USA). All images were measured using ImageJ software (Version 1.51j8, National Institutes of Health, Bethesda, MD, USA).

## 3. Results

### 3.1. The Level of APPL2 Decreases in the Major Organs of Aged Mice and Lack of APPL2 Leads to Premature Ageing of HUVECs

**Figure 1:**
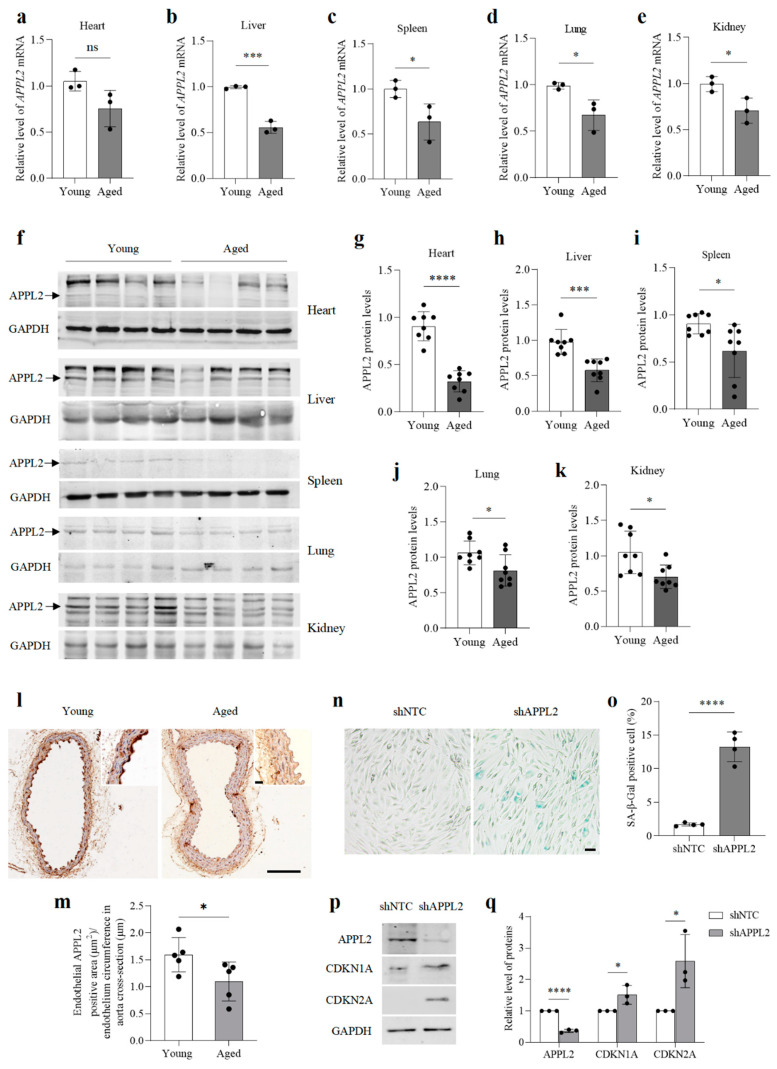
APPL2 levels decrease in the major organs of aged mice and APPL2 deficiency leads to premature ageing in HUVECs. (**a**–**k**), qRT-PCR (**a**–**e**), and Western blot (**f**–**k**) analyses of the major organs obtained from young (3 months) and aged (≥21 months) C57BL/6 mice. For (**a**–**e**), *n* = 3 mice per group. For (**f**–**k**), *n* = 8 mice per group. (**l**,**m**) Immunohistochemical staining of aortic APPL2 in young and aged C57BL/6 mice. *n* = 5 mice per group. Bar = 100 μm. Bar in zoomed figure = 20 μm. n,o, SA-β-gal staining of HUVECs (PD5) expressing shNTC, or shAPPL2. Bar = 30 μm. (**p**,**q**) Western blot analysis of APPL2, CDKN1A, and CDKN2A in HUVECs (PD5) expressing shNTC or shAPPL2. Three (**a**–**m**,**p**,**q**) or four (**n**,**o**) biologically independent experiments. Data are shown as mean ± s.d.; *p* values are derived from two-tailed unpaired Student’s *t*-tests; * *p* < 0.05, *** *p* < 0.001, **** *p* < 0.0001, ns means not significant. The bullets in the statistical graph represent the values for each sample per independent experiment.

To investigate whether APPL2 levels change during ageing, we compared the content of APPL2 in the major organs obtained from young and aged mice. Both the mRNA and protein levels of APPL2 were significantly reduced in the major organs of aged mice, including the liver, spleen, lungs, and kidneys, compared with those of young animals (Figure 1a–k). Although no significant changes were detected in the mRNA level of APPL2 in the hearts of aged mice, its protein level decreased. In addition, strong immunoreactivity for APPL2 was detected in the aortic endothelium of young mice but not in that of aged animals (Figure 1l,m), suggesting that the level of APPL2 decreases during ageing.

Then, we transduced HUVECs with Lenti-shAPPL2 to knock down APPL2 (Figure 1n). At population doubling (PD) five, these cells exhibited significant senescence-related phenotypes compared to non-targeted control cells, including increased SA-β-gal activity and enhanced levels of CDKN1A (p21) and CDKN2A (p16) (Figure 1n–q), suggesting that APPL2 might be involved in regulating ageing.

### 3.2. T04C9.1 Deficiency Reduces Longevity without Altering the Reproduction of C. elegans

To investigate the role of APPL2 in ageing, we searched for *C. elegans* APPL2 homologues and found that *T04C9.1* is sequentially similar to APPL2 in human (Q8NEU8), mouse (Q8K3G9), bat (G1P990), chicken (A0A8V0YBR8), and dog (A0A8I3MVM2) species (Supplementary Figure S1). The *T04C9.1* gene localizes on chromosome 3 and has 17 exons with an estimated 863 amino acids. We compared the BAR domain and Pleckstrin domain of *T04C9.1* with that of human APPL2. These regions share 21.53% and 31.03% homology with APPL2 at the amino acid level. 

**Figure 2:**
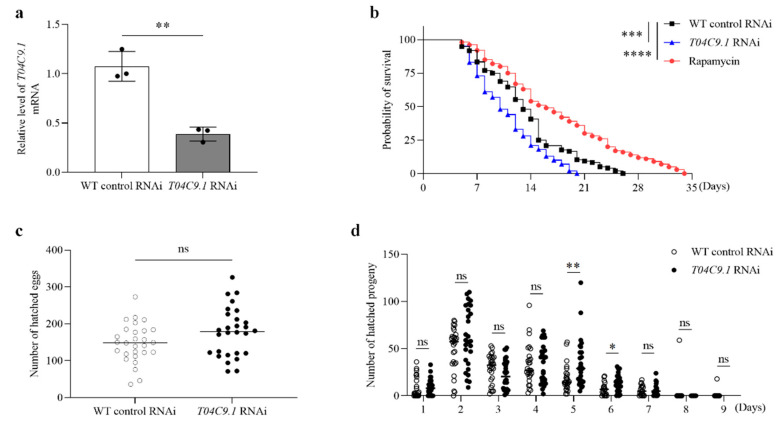
Effects of *T04C9.1* deficiency on longevity and reproduction of *C. elegans*. (**a**) qRT-PCR analysis of *T04C9.1* in nematodes fed RNAi bacteria targeting empty vector or *T04C9.1. n* = 1000 nematodes per group. (**b**) Survival curves of nematodes fed RNAi bacteria targeting empty vector, *T04C9.1*, or treated with rapamycin as a positive control. *n* = 100 nematodes per group. (**c**) Total number of hatched eggs laid per nematode. *n* = 28 nematodes per group. (**d**) Number of hatched eggs laid per nematode every day. *n* = 28 nematodes per group. Three biologically independent experiments. Data are shown as mean ± s.d.; *p* values are derived from two-tailed unpaired Student’s *t*-tests (**a**,**c**,**d**) or the Log-rank (Mantel–Cox) test (**b**); ** *p* < 0.01, *** *p* < 0.001, **** *p* < 0.0001, n.s. means not significant. The bullets in the statistical graph represent the values for each sample per independent experiment.

The level of *T04C9.1* in aged nematodes is significantly lower than that in young individuals (Supplementary Figure S2). To further examine the role of *T04C9.1* in regulating *C. elegans* longevity, we performed RNA interference (RNAi) to silence *T04C9.1* by feeding nematodes with bacteria expressing double-stranded RNA (dsRNA) against *T04C9.1.* qRT-PCR analysis revealed that the mRNA levels of *T04C9.1* were significantly decreased in these animals (Figure 2a and Supplementary Figure S3). Among the three different dsRNAs, dsRNA-3 is the most effective; thus, we used dsRNA-3 to knock down *T04C9.1* in our experiments. There was no statistically significant difference in the lifespan, motility, pharyngeal pumping, body bending frequency, total number of hatched eggs, and reproductive period between empty-vector treated and untreated nematodes (Supplementary Figure S4 and Supplementary Movies S1 and S2). We found that the knockdown of *T04C9.1* shortened the lifespan of nematodes (Figure 2b). *T04C9.1* knockdown (*T04C9.1* KD) did not alter the total number of hatched eggs and the reproductive period (Figure 2c,d), suggesting that *T04C9.1* deficiency reduces *C. elegans* longevity without altering their reproduction. Our results are consistent with a previous study showing that the absence of APPL2 did not impair reproductive function in mice [21].

### 3.3. T04C9.1 Deficiency Leads to Lipofuscin Pigment Accumulation, Lipid Deposition, and Mobility Decline in C. elegans

**Figure 3:**
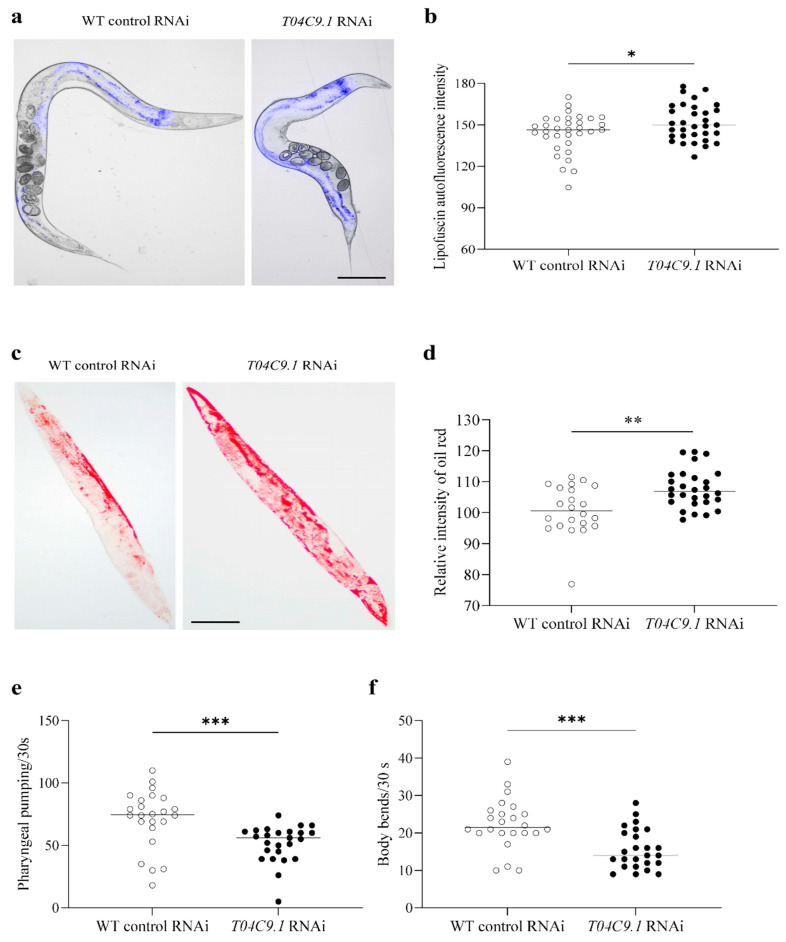
Effects of *T04C9.1* deficiency on lipofuscin accumulation, lipid deposition, and locomotor capacity in *C. elegans*. (**a**,**b**) Detection of lipofuscin autofluorescence in nematodes fed RNAi bacteria targeting empty vector or *T04C9.1. n* = 33 nematodes per group. Bar = 100 μm. (**c**,**d**) Oil red O staining of nematodes fed RNAi bacteria targeting empty vector or *T04C9.1. n* = 22 nematodes in WT control RNAi group, *n* = 28 nematodes in *T04C9.1* RNAi group. Bar = 100 μm. (**e**,**f**) Number of pumps (**e**) and body bends (**f**) monitored during a 30 s interval in nematodes fed RNAi bacteria targeting empty vector or *T04C9.1. n* = 24 nematodes in WT control RNAi group, *n* = 25 nematodes in *T04C9.1* RNAi group. Three biologically independent experiments. Data are shown as mean ± s.d.; *p* values are derived from two-tailed unpaired Student’s *t*-tests; * *p* < 0.05, ** *p* < 0.01, *** *p* < 0.001. The bullets in the statistical graph represent the values for each sample per independent experiment.

We next investigated whether *T04C9.1* deficiency causes age-associated alternations. Lipofuscin is an autofluorescent age pigment that accumulates in the intestinal cells of *C. elegans* and its content gradually increases throughout *C. elegans* adulthood [22]. The accumulation of lipofuscin is the most prominent age-associated alteration in *C. elegans*. *T04C9.1* KD significantly increased the lipofuscin deposition compared to the control nematodes (Figure 3a,b). Decreased longevity may be related to fat metabolism; therefore, we determined whether the lack of *T04C9.1* increases fat accumulation in vivo. As revealed by the quantification of oil red O staining, *T04C9.1* KD induced a significant increase in lipid deposition in *C. elegans* compared to that of control animals (Figure 3c,d). Another feature of ageing in *C. elegans* is characterized by a progressive decline in motor activity [22]. *T04C9.1*-KD nematodes exhibited a reduction in locomotion speed and travel distance as measured by video recordings of nematodes moving on an agar plate without food (Movies S3 and S4). Moreover, *T04C9.1* KD reduced the pharyngeal pumping and body bending frequency in *C. elegans* (Figure 3e,f). These results collectively showed that *T04C9.1* deficiency leads to premature ageing in *C. elegans*.

### 3.4. T04C9.1 Deficiency Increases the Stress Sensitivity and Reactive Oxygen Species (ROS) Levels in C. elegans

**Figure 4:**
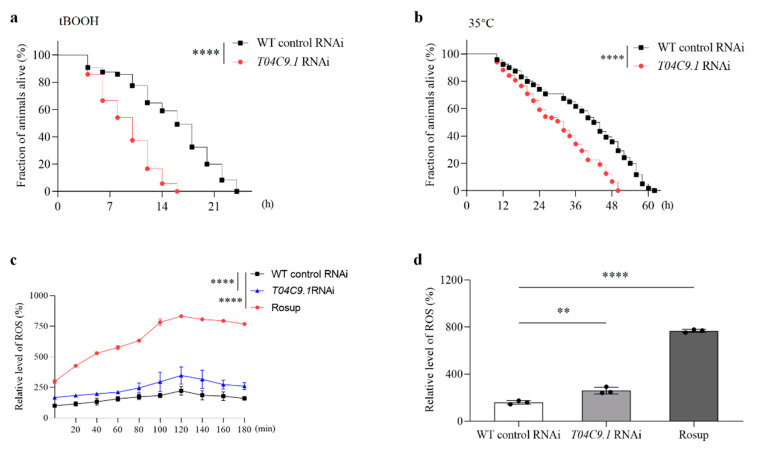
Effects of *T04C9.1* deficiency on stress resistance and ROS levels in *C. elegans.* (**a**,**b**) Probability of survival of nematodes fed RNAi bacteria targeting empty vector or *T04C9.1* and exposed to 6 mM tBOOH (**a**) or transferred to 35 °C (**b**). *n* = 120 nematodes per group. (**c**,**d**) ROS levels in nematodes fed RNAi bacteria targeting empty vector or *T04C9.1.* were determined by DCFH-DA assay. Data represent ROS levels measured every 20 min for 3 h (**c**) or measured at 3 h (**d**). *n* = 80 nematodes per group. Three biologically independent experiments. Data are shown as mean ± s.d.; *p* values are derived from the Log-rank (Mantel–Cox) test (**a**,**b**), two-way ANOVA (**c**), or one-way ANOVA (**d**) with Dunnett’s multiple comparisons tests; * *p* < 0.05, **** *p* < 0.0001. The bullets in the statistical graph represent the values for each sample per independent experiment.

Disruption of stress response inhibits lifespan extension in *C. elegans* [23]. Therefore, we investigated whether the lack of *T04C9.1* increased the stress sensitivity of nematodes. We evaluated the stress tolerance of *T04C9.1*-KD nematodes under two unfavourable conditions: exposure to the oxidant tert-butyl hydroperoxide (t-BuOOH) and high temperature. Age-synchronous WT and *T04C9.1*-KD nematodes were treated with 6 mM t-BuOOH (oxidative stress) or shifted to 35 °C (thermal stress). *T04C9.1* KD reduced the tolerance of nematodes to oxidative stress and thermal stress, which was reflected in the decrease in the maximum lifespan of nematodes by 33.33% and 19.35%, respectively (Figure 4a,b). 

ROS cause molecular damage that accumulates with age and are widely recognized as one of the primary causes of ageing and age-related diseases [24]. We found that the levels of ROS in *T04C9.1*-KD nematodes were higher when compared with WT animals (Figure 4c,d), indicating that *T04C9.1* deficiency led to intracellular ROS accumulation in *C. elegans*.

### 3.5. T04C9.1 Deficiency Decreases Autophagic Activity in C. elegans

**Figure 5:**
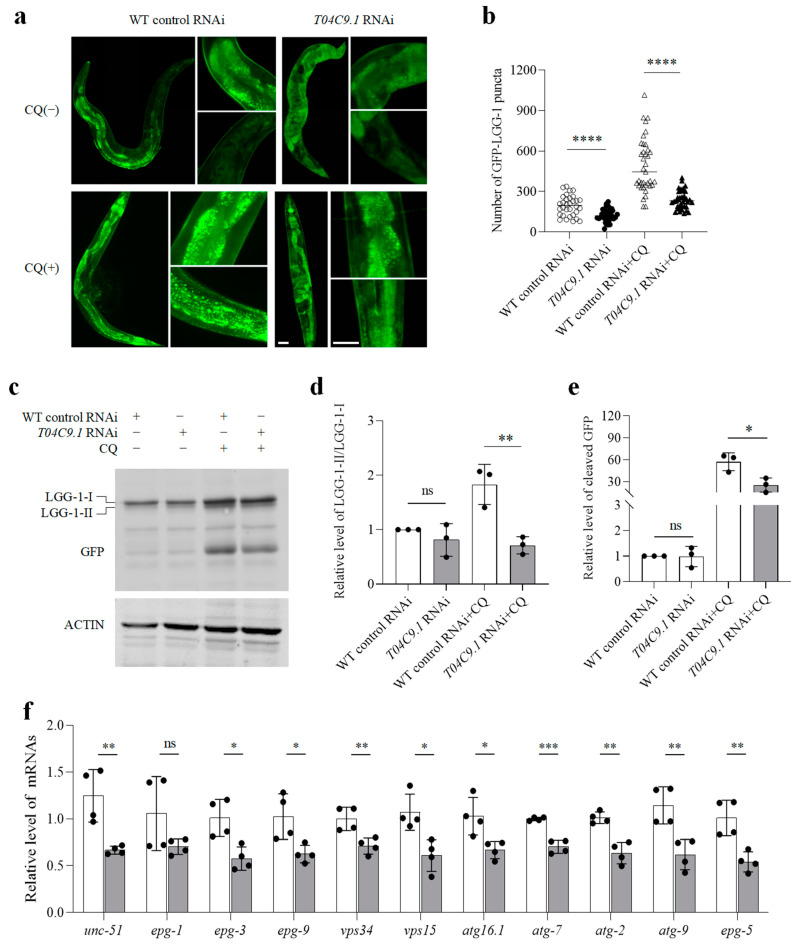
*T04C9.1* deficiency decreases autophagic activity in *C. elegans*. (**a**,**b**) Detection of GFP::LGG1 puncta in nematodes fed RNAi bacteria targeting empty vector or *T04C9.1* in the absence or presence of CQ (100 mM). *n* = 32 nematodes in WT control RNAi group, *n* = 35 nematodes in WT control RNAi + CQ group, *n* = 34 nematodes in *T04C9.1* RNAi group, and *n* = 37 nematodes in *T04C9.1* RNAi + CQ group. (**a**) The right column exhibits the partially enlarged images. Bar = 20 μm. Bar in zoomed figure = 50 μm. (**c**–**e**) Western blot analysis of LGG-1-II/LGG-1-I and free GFP in nematodes fed RNAi bacteria targeting empty vector or *T04C9.1* in the absence or presence of CQ (100 mM). *n* = 2000 nematodes per group. Three (**a**–**e**) or four (**f**) biologically independent experiments. Data are shown as mean ± s.d.; *p* values are derived from two-tailed unpaired Student’s *t*-tests; * *p* < 0.05, ** *p* < 0.01, *** *p* < 0.001, **** *p* < 0.0001, n.s. means not significant. The bullets in the statistical graph represent the values for each sample per independent experiment.

As disabled autophagy is a hallmark of ageing in organisms [25], we analysed whether a lack of *T04C9.1* affects autophagy in *C. elegans*. Nematodes expressing the autophagy marker GFP:LGG-1 had less puncta in their intestinal cells compared to that of WT nematodes (Figure 5a,b). The reduced number of GFP::LGG-1 puncta can be due to decreased autophagy induction or, alternatively, increased autophagosome degradation. To discriminate between these two possibilities, we treated nematodes with chloroquine (CQ) to block autophagosome degradation. CQ treatment resulted in the formation of enlarged GFP::LGG-1-positive punctate structures (Figure 5a), a phenotype indicative of defective autophagosomes [23]. In the presence of CQ, the accumulation of GFP::LGG-1 and free GFP protein in *T04C9.1*-KD nematodes was less than that of control animals (Figure 5c–e), suggesting that *T04C9.1* deficiency suppresses autophagic activity and that the reduced number of GFP::LGG-1 puncta seen in nematodes lacking *T04C9.1* is not due to enhanced autophagosome degradation in the late steps of autophagy. In addition, we performed qPCR analysis on a series of key genes involved in autophagy regulation and found that, except for *epg-1*, the expression of other autophagy-related genes detected in *T04C9.1*-KD nematodes was significantly reduced (Figure 5f). These results collectively demonstrated that *T04C9.1* deficiency caused autophagy decline in *C. elegans.*

### 3.6. T04C9.1 Deficiency Leads to Premature Ageing and Shortened Lifespan of C. elegans by Reducing Autophagy

To clarify whether the premature ageing caused by *T04C9.1* deficiency is due to decreased autophagy, we treated *T04C9.1*-KD nematodes with rapamycin (a well-known autophagy inducer) or fed them with bacteria expressing double-stranded RNA (dsRNA) for *let-363* (*C. elegans* Tor, orthologous to mechanistic target of rapamycin, mTOR). We found that rapamycin increased the number of GFP::LGG-1 puncta in *T04C9.1*-KD nematodes (Supplementary Figure S5a,b). Western blotting confirmed increased LGG-1 protein levels and LGG-1 processing in rapamycin-treated *T04C9.1*-KD nematodes (Supplementary Figure S5c,d), suggesting that rapamycin enhances autophagic activity in *T04C9.1*-KD nematodes. Similar to rapamycin treatment, we observed enhanced autophagic activity in *T04C9.1*-KD nematodes subjected to RNAi against *let-363* (Supplementary Figure S5e–h). 

**Figure 6:**
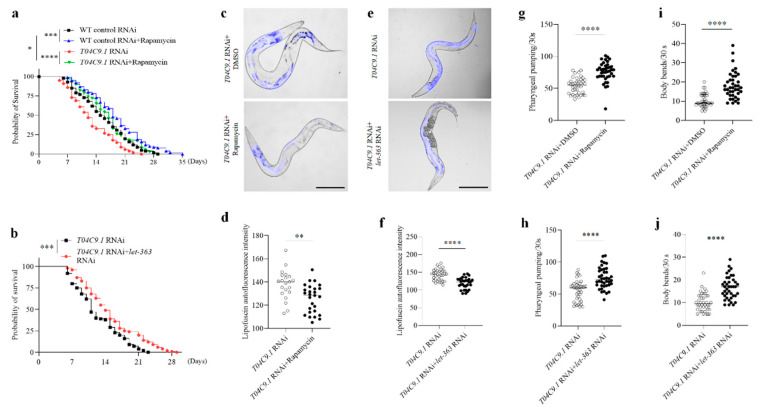
Effects of rapamycin or inhibition of *let-363* on longevity, lipofuscin accumulation, and motility of *T04C9.1*-deficient *C. elegans.* (**a**,**b**) Survival curves of untreated nematodes, nematodes fed with RNAi bacteria targeting *T04C9.1*, or empty vector in the absence or presence of rapamycin (100 μM) (**a**) or fed with RNAi bacteria targeting *T04C9.1* alone or in combination with RNAi bacteria targeting *let-363* (**b**). *n* = 120 nematodes per group. (**c**–**f**) Detection of lipofuscin autofluorescence in nematodes fed with RNAi bacteria targeting *T04C9.1* in the absence or presence of rapamycin (100 μM) (**c**,**d**) or fed with RNAi bacteria targeting *T04C9.1* alone or in combination with RNAi bacteria targeting *let-363* (**e**,**f**). *n* = 21 nematodes in *T04C9.1* RNAi group (**c**,**d**), *n* = 25 nematodes in *T04C9.1* RNAi + rapamycin group, *n* = 34 nematodes in *T04C9.1* RNAi group (**e**,**f**), and *n* = 31 nematodes in *T04C9.1* RNAi + *let-363* RNAi group. Bar = 100 μm. g,h. Number of pumps monitored during a 30 s interval in nematodes fed with RNAi bacteria targeting *T04C9.1* in the absence or presence of rapamycin (100 μM) (**g**) or fed with RNAi bacteria targeting *T04C9.1* alone or in combination with RNAi bacteria targeting *let-363* (**h**). *n* = 40 nematodes per group. (**i**,**j**) Number of body bends monitored during a 30 s interval in the absence or presence of rapamycin (100 μM) (**i**) or fed with RNAi bacteria targeting *T04C9.1* alone or in combination with RNAi bacteria targeting *let-363* (**j**). *n* = 40 nematodes per group. Three biologically independent experiments. Data are shown as mean ± s.d.; *p* values are derived from the Log-rank (Mantel–Cox) test (**a**,**b**) or two-tailed unpaired Student’s *t*-tests (**d**,**f**–**j**); * *p* < 0.05, ** *p* < 0.01, *** *p* < 0.001, **** *p* < 0.0001. The bullets in the statistical graph represent the values for each sample per independent experiment.

Rapamycin or inhibition of *let-363* activated autophagy in *T04C9.1*-KD nematodes and reversed the shortened lifespan (Figure 6a,b), age pigment accumulation (Figure 6c–f), and mobility decline (Figure 6g–j), suggesting that the decreased autophagy underlies premature ageing caused by *T04C9.1* deficiency. 

**Figure 7:**
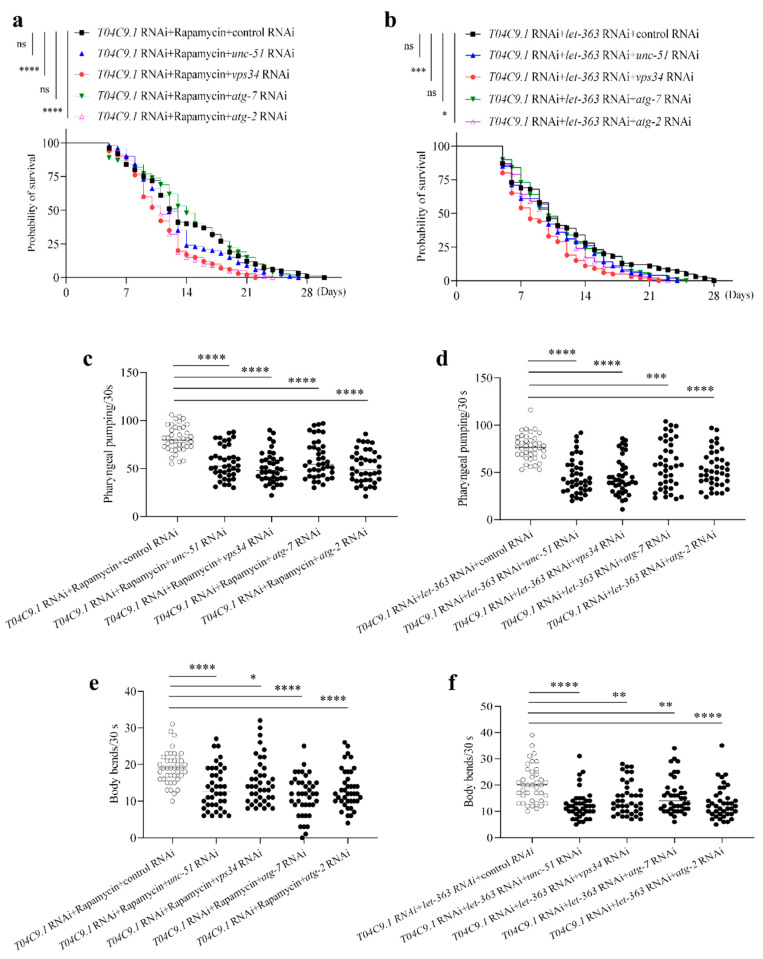
Knocking down the autophagy-associated gene reversed the effects of rapamycin or inhibition of *let-363* on longevity and motility in *T04C9.1*-KD *C. elegans.* (**a**,**b**) Survival curves of nematodes fed with RNAi bacteria targeting *T04C9.1* in combination with RNAi bacteria targeting empty vector, *unc-51*, *vps34*, *atg-7*, or *atg-2* in the presence of rapamycin (100 μM) (**a**) or fed with RNAi bacteria targeting *T04C9.1* and *let-363* in combination with RNAi bacteria targeting empty vector, *unc-51*, *vps34*, *atg-7*, or *atg-2* (**b**). *n* = 100 nematodes per group. (**c**,**d**) Number of pumps monitored during a 30 s interval in nematodes fed with RNAi bacteria targeting *T04C9.1* in combination with RNAi bacteria targeting empty vector, *unc-51*, *vps34*, *atg-7*, or *atg-2* in the presence of rapamycin (100 μM) (**c**) or fed with RNAi bacteria targeting *T04C9.1* and *let-363* in combination with RNAi bacteria targeting empty vector, unc-51, *vps34*, *atg-7*, or *atg-2* (**d**). *n* = 40 nematodes per group. (**e**,**f**) Number of body bends monitored during a 30 s interval in nematodes fed with RNAi bacteria targeting *T04C9.1* in combination with RNAi bacteria targeting empty vector, *unc-51*, *vps34*, *atg-7*, or *atg-2* in the presence of rapamycin (100 μM) (**e**) or fed with RNAi bacteria targeting *T04C9.1* and *let-363* in combination with RNAi bacteria targeting empty vector, *unc-51*, *vps34*, *atg-7*, or *atg-2* (**f**). *n* = 40 nematodes per group. Three biologically independent experiments. Data are shown as mean ± s.d.; *p* values are derived from the Log-rank (Mantel–Cox) test (**a**,**b**) or one-way ANOVA with Dunnett’s multiple comparisons test (**c**–**f**); * *p* < 0.05, ** *p* < 0.01, *** *p* < 0.001, **** *p* < 0.0001, n.s. means not significant. The bullets in the statistical graph represent the values for each sample per independent experiment.

To support this notion, we further investigated whether genetic inhibition of autophagy by RNAi against four core autophagy components (*unc-51*, *vps34*, *atg-7* and *atg-2*) affects the lifespan of *T04C9.1*-KD nematodes treated with rapamycin or subjected to RNAi against *let-363*. The results of qRT-PCR confirmed that the use of multiple RNAi did not negate the effects of these RNAi treatments, that is, one RNAi did not compensate for the effect of another and all RNAi worked (Supplementary Figure S6). We found that knocking down *vps34* or *atg2* fully curtailed lifespan extension triggered by rapamycin or inhibition of *let-363* in *T04C9.1*-KD nematodes (Figure 7a,b). Although knocking down *unc-51* or *atg-7* did not significantly limit the lifespan extension in *T04C9.1*-KD nematodes treated with rapamycin or *let-363* RNAi, the lack of either of these four core autophagy components significantly accelerated mobility impairment (Figure 7c–f) and age pigment accumulation (Supplementary Figure S7). These results collectively demonstrated that *T04C9.1* deficiency leads to premature ageing and shortened lifespan of *C. elegans* by reducing autophagy.

## 4. Discussion

The risk of many chronic diseases, including cardiovascular diseases, neurodegenerative diseases, and cancer, increases exponentially with age. Research aimed at understanding age-related molecular changes can help uncover new biomarkers of ageing and be beneficial in reducing these risks. APPL2, one of the APPL isoforms, interacts with a variety of receptors and signalling molecules in cells [6]. Unlike APPL1, the physiological functions of APPL2 are less clear. It should not be ignored that human studies have linked the genetic variations in APPL2 to increased cardiovascular risk, obesity, and the incidence of non-alcoholic fatty liver disease [26,27,28]. Deletion of APPL2 leads to glucose intolerance and defective glucose-stimulated insulin secretion [9]. When challenged with lipopolysaccharides, the lack of APPL2 in mice makes them more sensitive to endotoxin shock and increases the production of serum proinflammatory cytokines [29]. Since ageing is considered a systemic inflammatory process that raises the risk of various diseases such as cardiovascular disease and type II diabetes, coupled with our observation that APPL2 levels decrease with age in major mouse tissues, it is of interest to investigate the relationship between APPL2 and ageing. We found that knocking down APPL2 induced premature senescence in young HUVECs, suggesting a potential role for APPL2 in ageing.

Breath-taking insights into the underlying regulatory mechanisms of many critical aspects of the human ageing process have been gained using model organisms. Compared with other animal models, *C. elegans* has a small body size, short lifespan, completely sequenced genome, and more than 65% of the genes associated with human diseases, making this organism an ideal living system for the study of ageing and ageing-related diseases [30]. In the current study, we found that, similar to APPL2, the *C. elegans* APPL2 homologue *T04C9.1* declines with age. Knocking down *T04C9.1* shortened its lifespan and accelerated the age-associated deterioration in nematodes. In addition, we found that *T04C9.1*-KD nematodes were significantly more sensitive to oxidative and thermal stress. One of the signs of organismal ageing is the decline in adaptive stress response, which leads to a variety of age-related diseases [31]. Thus, our results collectively demonstrated that the lack of *T04C9.1* accelerates ageing and shortens the lifespan of *C. elegans*.

During the past few years, many conserved signalling pathways regulating ageing and lifespan have been identified using model organisms including yeast, worms, flies, and rodents. These signalling pathways include, but are not limited to, insulin/IGF-1, caloric restriction, TOR, Sirtuin 1, and CD44 [14,32,33]. Notably, among the numerous changes in ageing-related signalling pathways, altered autophagy has emerged as a convergent downstream mechanism of ageing across diverse species [32,33,34]. Since normal and pathological ageing is often associated with reduced autophagic potential, disabled autophagy is regarded as one of the hallmarks of ageing [25]. Pharmacological or genetic manipulations that increase the lifespan of model organisms often enhance autophagy activity and, when autophagy is inhibited, these manipulations lose their regulatory effects on lifespan [32,33,34]. Our study reveals, for the first time, that *T04C9.1* deficiency leads to decreased autophagy in *C. elegans.* This evidence prompts us to further investigate whether *T04C9.1* deficiency-induced premature ageing and shortened lifespan can be attributed to decreased autophagy. Our results confirmed the speculation that decreased autophagy leads to *T04C9.1* deficiency-induced ageing and shortened lifespan and uncovered the bridging role of *T04C9.1* between autophagy decline and ageing. Surprisingly, the lack of *unc-51* or *atg-7* negated the health effects of rapamycin or *let-363* RNAi but did not inhibit the effects of rapamycin or *let-363* RNAi on nematode lifespan. A possible explanation for this might be that *unc-51* or *atg-7* would be involved in other functions than autophagy, which affects the lifespan of nematodes. It has been reported that knocking down *unc-51* or *atg-7* does not reduce the lifespan of daf-2 mutants but, rather, tends to extend their lifespan [35], which supports our speculation.

In the present study, we found that the protein levels of APPL2 in the major organs of mice, including heart, liver, spleen, lung, and kidney, decreased in aged mice when compared to young mice. Our findings contradict the results of a previous study, which has shown that APPL2 content is elevated in the skeletal muscles of old rats [36]. This discrepancy may firstly be due to the low quality of data in this previous study, as reflected in the unclear APPL2 and endogenous control GAPDH bands in Western blot images, making the conclusion unconvincing. Secondly, these different results may be caused by different tissues and conditions. For example, systemic and hepatocyte-specific knockout of APPL2 has different or even opposite effects on the inflammatory responses triggered by different factors. When exposed to LPS, APPL2 knockout mice exhibited more severe endotoxic shock symptoms, accompanied by elevated levels of pro-inflammatory cytokines [29]. However, hepatocyte-specific knockout of APPL2 enhances the resistance of mice to high-fat diet-induced inflammation in liver tissues [15]. Such observations support this possibility.

In conclusion, this study provides evidence that the levels of APPL2 decrease with age and indicates that *T04C9.1* is necessary for maintaining autophagic activity during ageing. Lacking *T04C9.1* not only causes age-related alternations in young nematodes, making them more susceptible to stress but also significantly shortens the lifespan of these animals. Increasing evidence has uncovered a negative correlation between decreased autophagy and longevity. However, the molecular basis for why autophagic activity declines with age in many organisms is far from clear. Our study shows, for the first time, that the lack of *T04C9.1* during nematode ageing may help explain age-dependent autophagy decline, thereby shortening the animal’s lifespan and causing premature ageing. Further studies focusing on the mechanisms by which *T04C9.1* regulates autophagy will open up novel avenues for healthy ageing and longevity. PPI analysis of the interactions between APPL2 and 222 autophagy-related proteins revealed that APPL2 only interacts with RAB5A (Supplementary Figure S8), providing clues for further elucidation of the underlying mechanisms. Previous studies have shown that RAB5A binds to the PtdIns3K complex and promotes autophagy activity [37,38]. Molecular docking analysis showed that the affinity energy of the APPL2-PtdIns3K complex is −5.4 kcal/mol, indicating that they bind tightly (Supplementary Figure S9). The binding stability of the APPL2-PtdIns3K complex is supported by the two terminal amino acids of PIK3R4, which is stabilized by multiple hydrogen bonds. Therefore, we speculate that APPL2 and its homologue may regulate autophagy by interacting with the RAB5A and PtdIns3K complex, which will be elaborated in detail in the future.

## Data Availability

The data that support the findings of this study are available from the corresponding author upon reasonable request.

## Supplementary Materials

The following supporting information can be downloaded at https://www.mdpi.com/article/10.3390/genes15060659/s1: Figure S1: Identification of *C. elegans* APPL2 homologue; Figure S2: qRT-PCR analysis of the levels of T04C9.1 in young and aged *C. elegans*; Figure S3: qRT-PCR analysis of *T04C9.1* in nematodes fed RNAi bacteria targeting empty vector or *T04C9.1*; Figure S4: There was no statistically difference in the lifespan, pharyngeal pumping, body bending frequency, total number of hatched eggs, and reproductive period between empty-vector treated and untreated nematodes; Figure S5: Rapamycin treatment or knockdown of *let-363* activates autophagy in *T04C9.1*-KD *C. elegans*; Figure S6: The use of multiple RNAi does not negate the effects of these RNAi treatments; Figure S7: Knocking down autophagy-associated gene reversed the effects of rapamycin or inhibition of *let-363* on lipofuscin pigment accumulation in *T04C9.1*-KD *C. elegans*; Figure S8: Interaction network of APPL2 and 222 autophagy-related proteins; Figure S9: Structure modelling and molecular docking of APPL2 with PtdIns3K complex. Table S1: Detailed list of materials used in this study; Table S2: RT-PCR primer sequences. Movie S1: Motility vigour of the WT group nematode; Movie S2: Motility vigour of the WT control RNAi group nematode; Movie S3: Motility vigour of the WT control RNAi group nematode; Movie S4: Motility vigour of the *T04C9.1* RNAi group nematode.
