# Supplementary material for: Lack of T04C9.1, the Homologue of Mammalian APPL2, Leads to Premature Ageing and Shortens Lifespan in Caenorhabditis elegans
Source: Genes (Basel). 2024 May 22;15(6):659. doi: 10.3390/genes15060659 (PMC11202736; doi:10.3390/genes15060659)
Supplement: Supplementary file 1 [file genes-15-00659-s001.zip › genes-2977712-supplementary.pdf]

Supplementary information

Lack of T04C9.1, the Homologue of Mammalian APPL2, Leads to Premature Ageing and Shortens Lifespan in Caenorhabditis elegans

Zirui Li [†], Zhiqiang Chen [†], Lianghao Zhao, Jiaqi Sun, Lin Yin, Yuwei Jiang, Xiaotong Shi, Ziye Song and Lu Zhang [*]

College of Bioengineering, Henan University of Technology, 100 Lianhua Street, High-Tech Zone, Zhengzhou 450001, China

* Correspondence: zhanglu@haut.edu.cn; Tel.: +86-371-67756513

[†] These authors contributed equally to this work.

Supplementary Figure S1. Identification of *C. elegans* APPL2 homologue.

Supplementary Figure S2. qRT-PCR analysis of the levels of *T04C9.1* in young and aged *C. elegans*.

Supplementary Figure S3. qRT-PCR analysis of *T04C9.1* in nematodes fed RNAi bacteria targeting empty vector or *T04C9.1*.

Supplementary Figure S4. There was no statistically difference in the lifespan, pharyngeal pumping, body bending frequency, total number of hatched eggs, and reproductive period between empty-vector treated and untreated nematodes.

Supplementary Figure S5. Rapamycin treatment or knockdown of *let-363* activates autophagy in *T04C9.1*-KD *C. elegans.*

Supplementary Figure S6. The use of multiple RNAi does not negate the effects of these RNAi treatments.

Supplementary Figure S7. Knocking down autophagy-associated gene reversed the effects of rapamycin or inhibition of *let-363* on lipofuscin pigment accumulation in *T04C9.1*-KD *C. elegans.*

Supplementary Figure S8. Interaction network of APPL2 and 222 autophagy-related proteins.

Supplementary Figure S9. Structure modeling and molecular docking of APPL2 with PI3KC3 complex.

Supplementary Figure S1

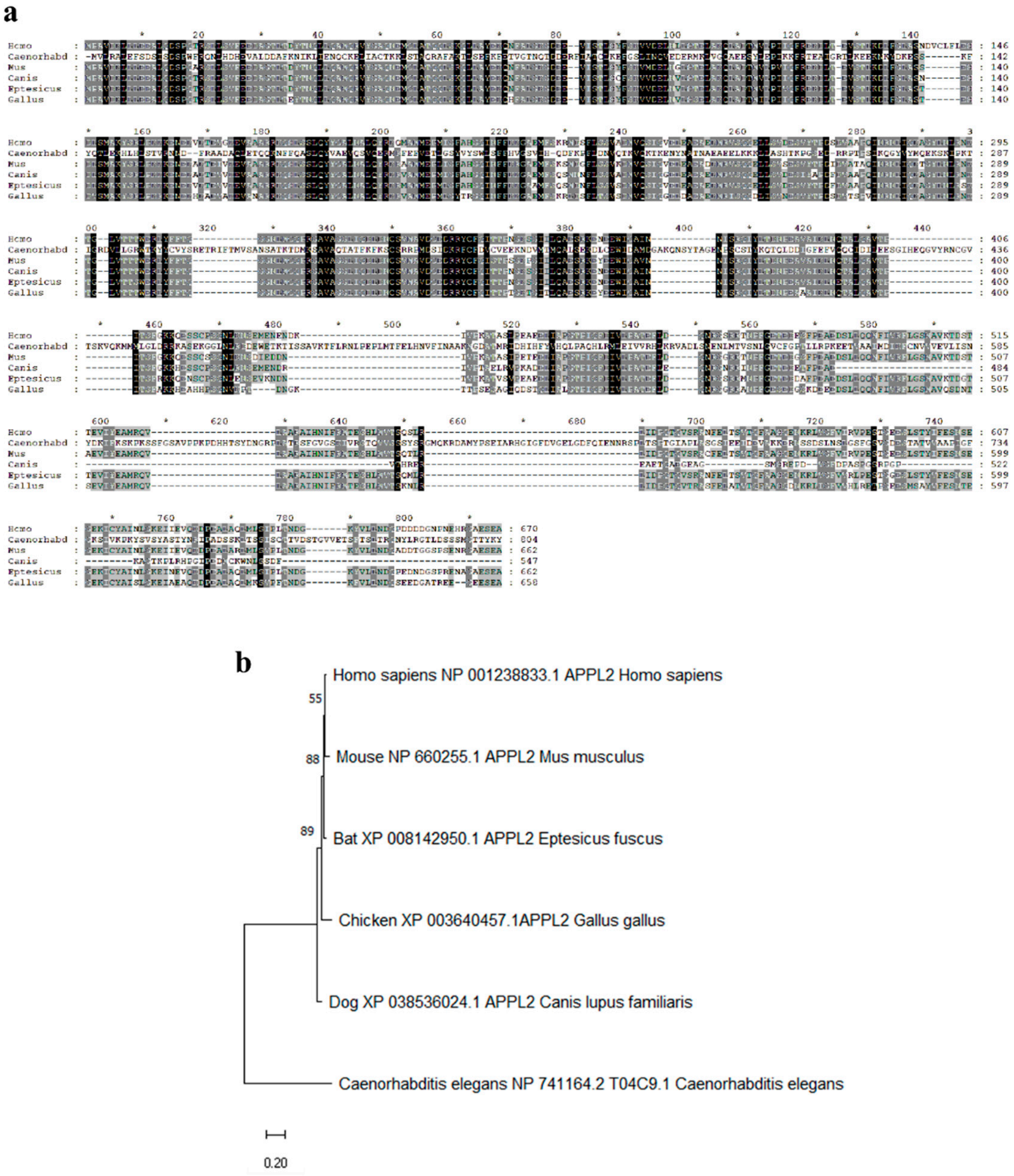

Supplementary Figure S1. Identification of *C. elegans* APPL2 homologue

a, Amino-acid alignment of the N-terminal regions of APPL2 homologues from several species (Homo sapiens; Mouse; Bat; Chicken; Dog; Caenorhabditis elegans) **b**, Phylogenetic tree generated by Mega11 showing that NP_741164.2 is similar to Homo sapiens NP_001238833.1, and Mouse NP_660255.1.

Supplementary Figure S2

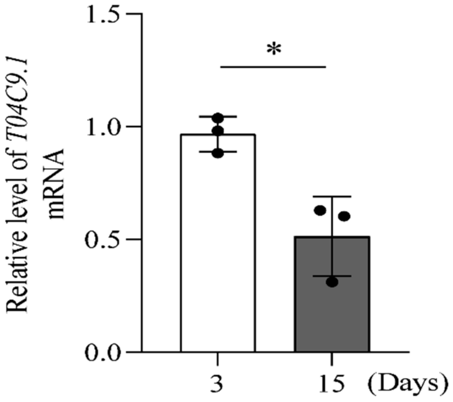

Supplementary Figure S2. qRT-PCR analysis of the levels of T04C9.1 in young and aged *C. elegans*. n = 1000 nematodes per group. Three biologically independent experiments. Data are shown as mean ± s.d.; *P* values are derived from Two-tailed unpaired Student's *t*-tests.

Supplementary Figure S3

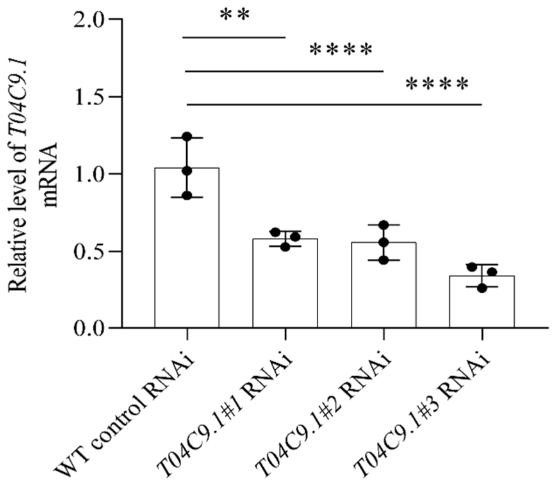

Supplementary Figure S3. qRT-PCR analysis of *T04C9.1* in nematodes fed RNAi bacteria targeting empty vector or *T04C9.1*. n = 1000 nematodes per group. Three biologically independent experiments. Data are shown as mean ± s.d.; *P* values are derived from Two-tailed unpaired Student's *t*-tests.

Supplementary Figure S4

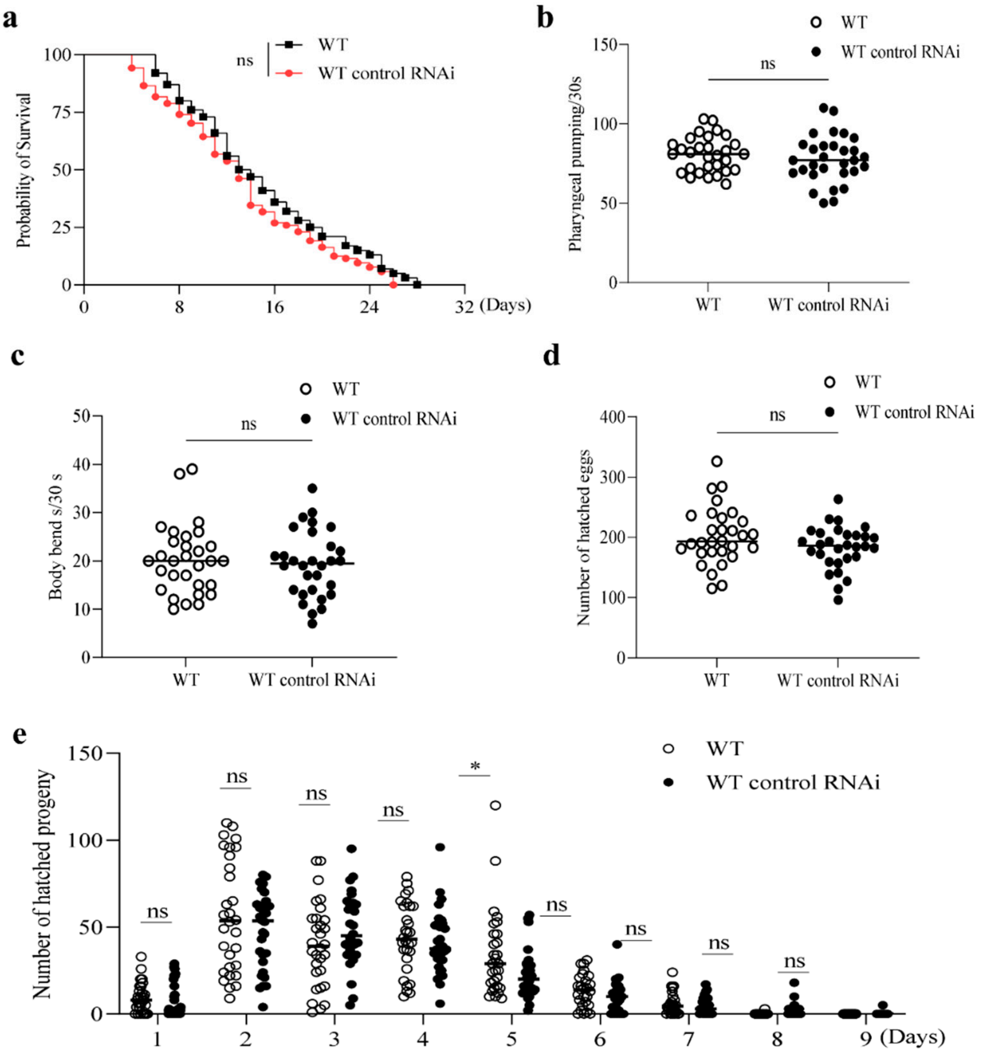

Supplementary Figure S4. There was no statistically difference in the lifespan, pharyngeal pumping, body bending frequency, total number of hatched eggs, and reproductive period between empty-vector treated and untreated nematodes.

a, Survival curves of empty-vector treated and untreated nematodes. n = 100 nematodes per group. **b**,**c**, Number of pumps (**b**) and body bends (**c**) monitored during a 30 s interval in nematodes whether or not fed RNAi bacteria targeting empty vector. n = 30 nematodes in WT group, n = 30 nematodes in WT control RNAi group. **d**, Total number of hatched eggs of laid per nematode. n = 30 nematodes per group. **e**, Number of hatched eggs laid per nematode every day. n = 30 nematodes per group. Three biologically independent experiments. Data are shown as mean ± s.d.; *P* values are derived from Two-tailed unpaired Student's *t*-tests (**b**, **c**, **d, e**) or Log-rank (Mantel-Cox) test (**a**).

Supplementary Figure S5

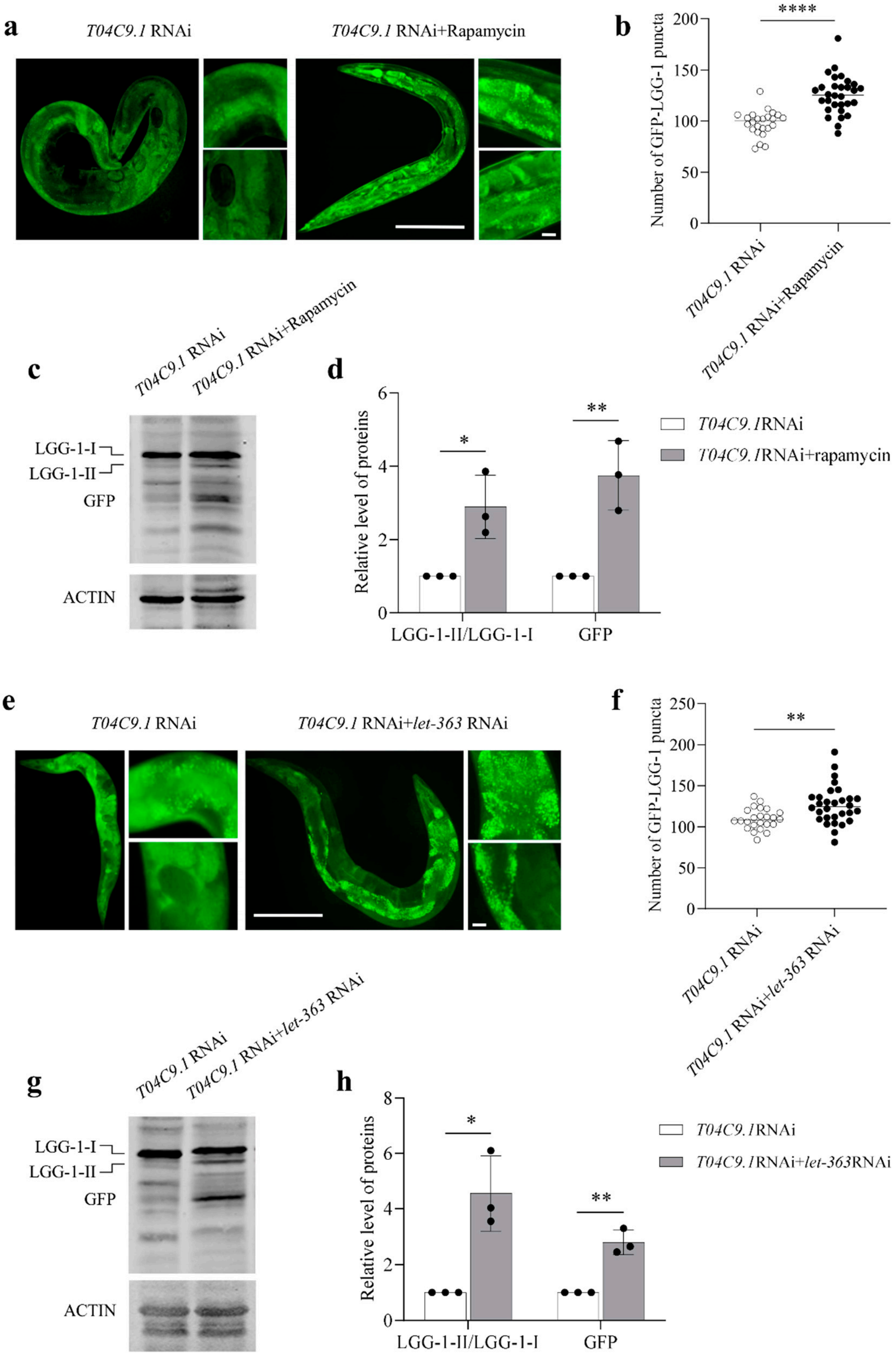

Supplementary Figure S5. Rapamycin treatment or knockdown of *let-363* activates autophagy in *T04C9.1*-KD *C. elegans.*

a,**b**, Detection of GFP::LGG1 puncta in nematodes fed with RNAi bacteria targeting *T04C9.1* in the absence or presence of rapamycin (100 μM). n = 24 nematodes in *T04C9.1* RNAi group, n = 29 nematodes in *T04C9.1* RNAi + rapamycin group. Bar = 100 μm. Bar in zoomed figure = 20 μm. **c**,**d**, Western blot analysis of LGG-1-II/LGG-1-I and free GFP in nematodes fed with RNAi bacteria targeting *T04C9.1* in the absence or presence of rapamycin (100 μM). n = 2000 nematodes per group. **e**,**f**, Detection of GFP::LGG1 puncta in nematodes fed with RNAi bacteria targeting *T04C9.1* alone or in combination with RNAi bacteria targeting *let-363*. n = 24 nematodes in *T04C9.1* RNAi group, n = 30 nematodes in *T04C9.1* RNAi + *let-363* RNAi group. Bar = 100 μm. Bar in zoomed figure = 20 μm. **g**,**h**, Western blot analysis of LGG-1-II/LGG-1-I and free GFP in nematodes fed with RNAi bacteria targeting *T04C9.1* alone or in combination with RNAi bacteria targeting *let-363*. n = 2000 nematodes per group. Three biologically independent experiments. Data are shown as mean ± s.d.; *P* values are derived from Two-tailed unpaired Student's *t*-tests.

Supplementary Figure S6

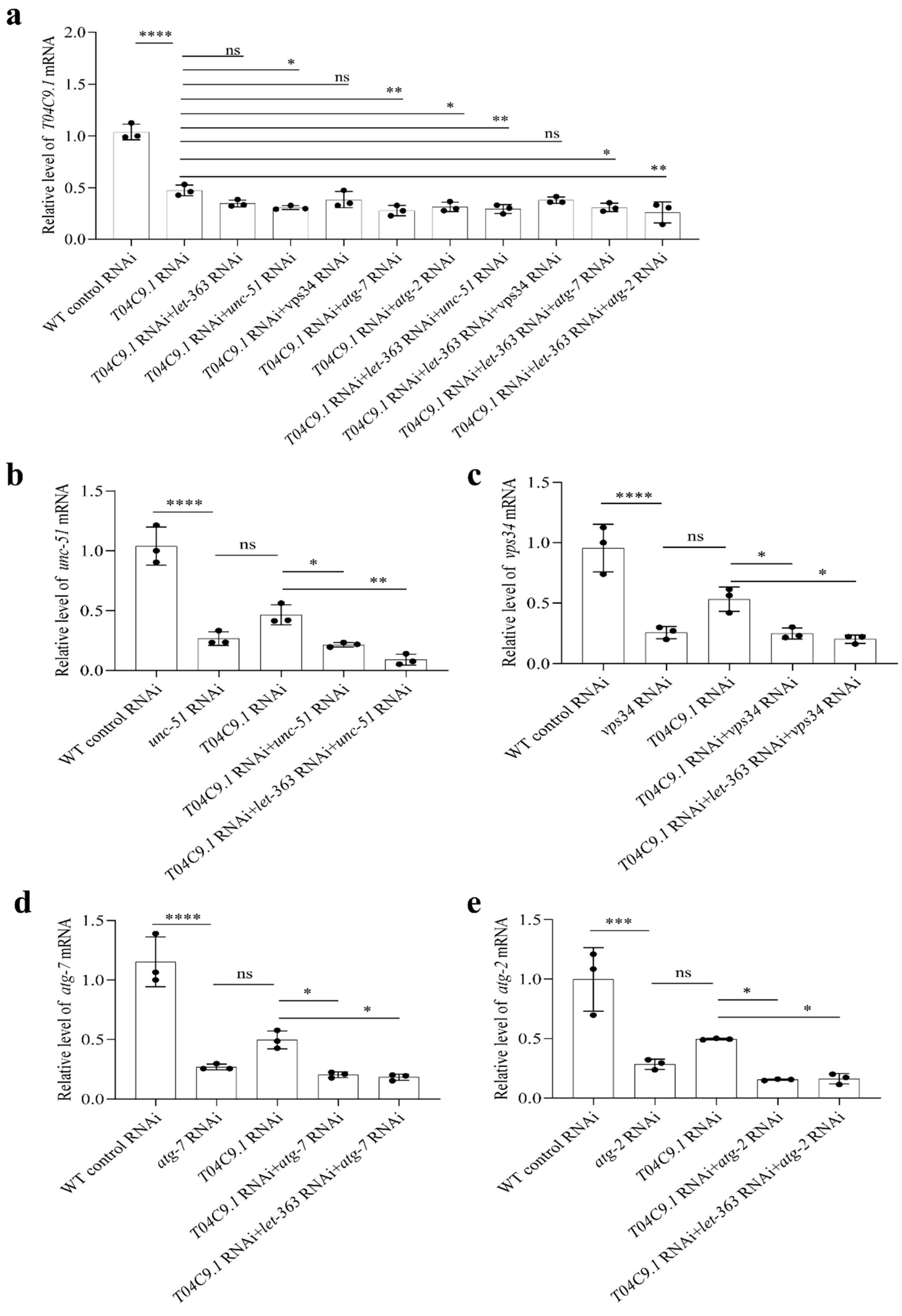

Supplementary Figure S6. The use of multiple RNAi does not negate the effects of these RNAi treatments.

a, qRT-PCR analysis of *T04C9.1* in nematodes fed RNAi bacteria targeting empty vector or different genes as indicated in figures. **b**-**e**, qRT-PCR analysis of *unc-51* (**b**), *vps34* (**c**), *atg-7* (**d**) or *atg-2* (**e**) in nematodes fed RNAi bacteria targeting empty vector or different genes as indicated in figures.

Supplementary Figure S7

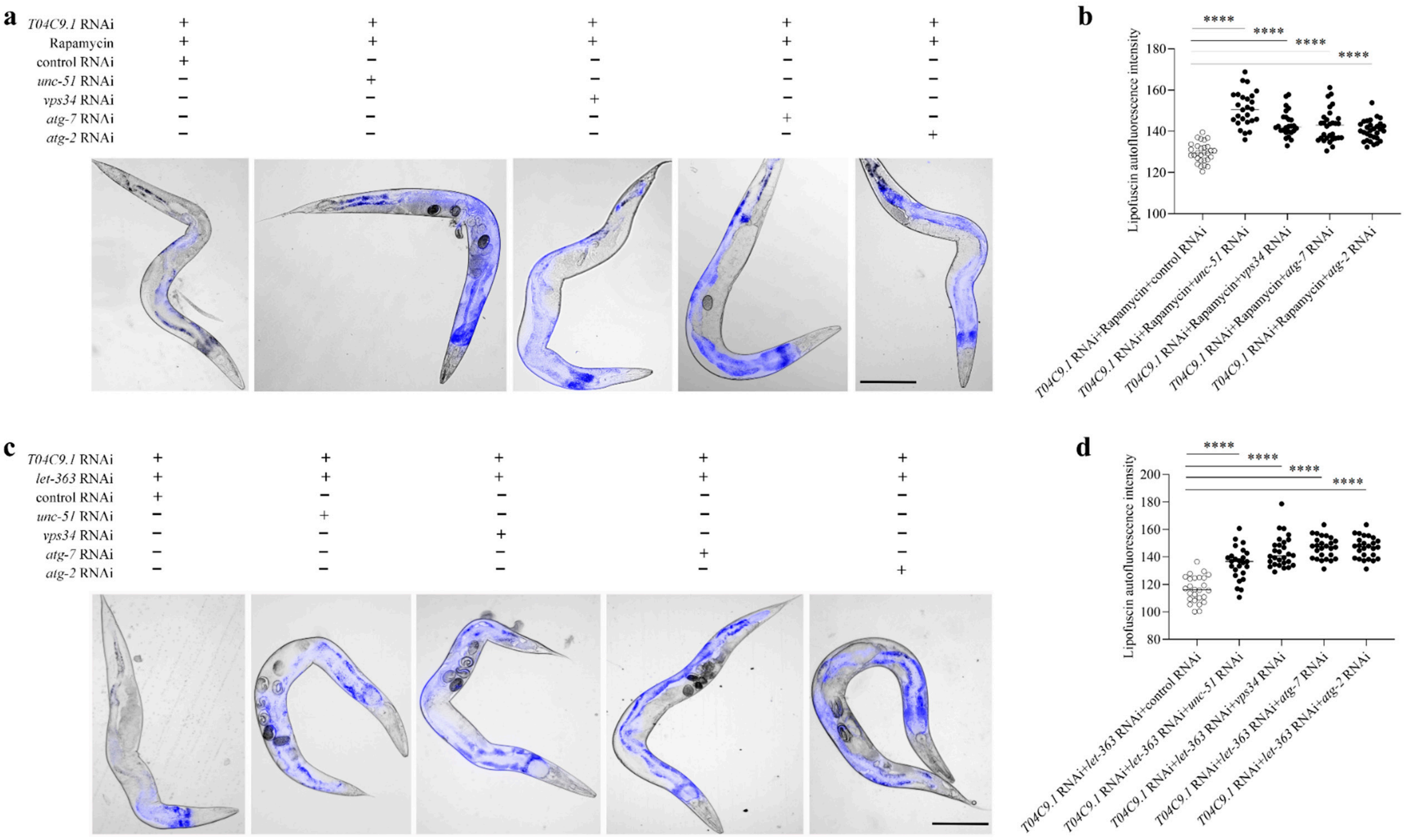

Supplementary Figure S7. Knocking down autophagy-associated gene reversed the effects of rapamycin or inhibition of *let-363* on lipofuscin pigment accumulation in *T04C9.1*-KD *C. elegans*.

a,**b**, Detection of lipofuscin autofluorescence of nematodes fed with RNAi bacteria targeting *T04C9.1* in combination with RNAi bacteria targeting empty vector, *unc-51*, *vps34*, *atg-7* or *atg-2* in the presence of rapamycin (100 μM). n = 29 nematodes in *T04C9.1* RNAi + rapamycin + control RNAi group, n = 26 nematodes in *T04C9.1* RNAi + rapamycin + *unc-51* RNAi group, n = 26 nematodes in *T04C9.1* RNAi + rapamycin + *vps34* RNAi group, n = 30 nematodes in *T04C9.1* RNAi + rapamycin + *atg-7* RNAi group, n = 30 nematodes in *T04C9.1* RNAi + rapamycin + *atg-2* RNAi group. Bar = 100 μm.

c,**d**, Detection of lipofuscin autofluorescence of nematodes fed with RNAi bacteria targeting *T04C9.1* and *let-363* in combination with RNAi bacteria targeting empty vector, *unc-51*, *vps34*, *atg-7* or *atg-2*. n = 26 nematodes in *T04C9.1* RNAi + *let-363* RNAi + control RNAi group, n = 25 nematodes in *T04C9.1* RNAi + *let-363* RNAi + *unc-51* RNAi group, n = 28 nematodes in *T04C9.1* RNAi + *let-363* RNAi + *vps34* RNAi group, n = 26 nematodes in *T04C9.1* RNAi + *let-363* RNAi + *atg-7* RNAi group, n = 29 nematodes in *T04C9.1* RNAi + *let-363* RNAi + *atg-2* RNAi group. Three biologically independent experiments. Data are shown as mean ± s.d.; *P* values are derived from one-way ANOVA with Dunnett's multiple comparisons test.

Supplementary Figure S8

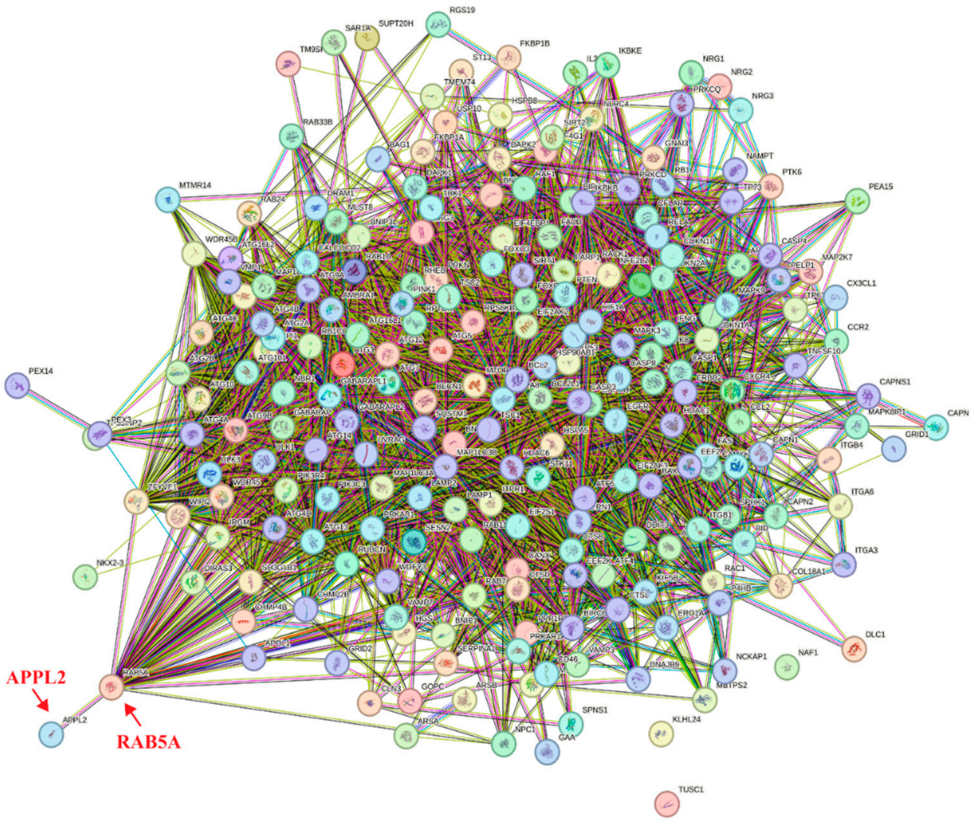

Supplementary Figure S8. Interaction network of APPL2 and 222 autophagy-related proteins. Interaction network diagram of APPL2 with 222 autophagy-related proteins using the STRING database. Each node represents all the proteins produced by a single, protein-coding gene locus. The type of interaction is indicated by edge colour—green: text mining, red: experiments, dark blue: gene co-occurrence, yellow orange: pathway databases, light blue: protein homology, dark blue: gene co-occurrence, black: co-expression, light green: gene neighbourhoods, pink: gene fusions.

Supplementary Figure S9

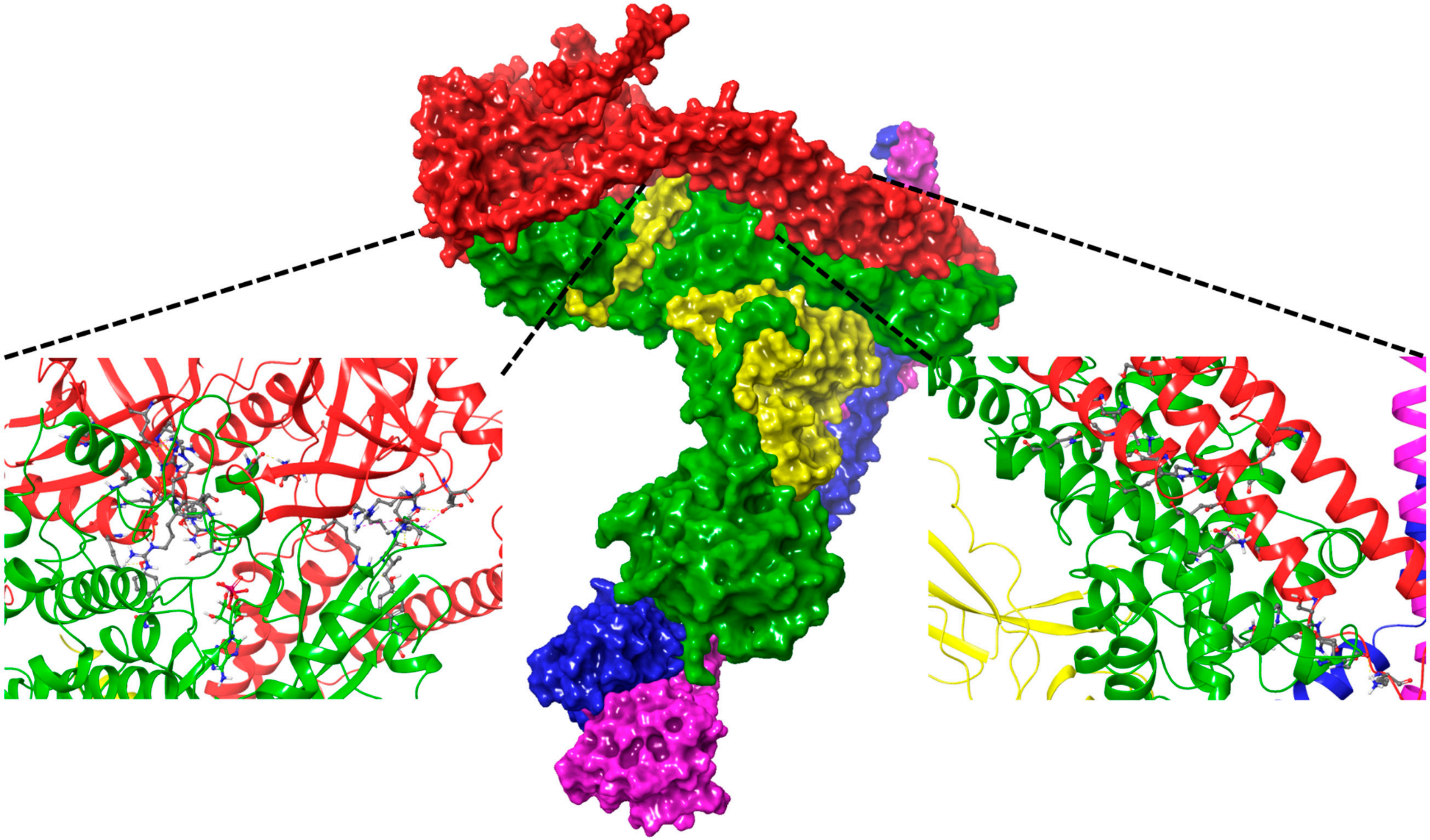

Supplementary Figure S9. Structure modeling and molecular docking of APPL2 with PI3KC3 complex. In order to study the binding mode between APPL2 and PtdIns3K complex, We constructed the APPL2 structure using Alphafold2. The stereo-chemistry of the best model was analyzed by using SAVES V6.0 (https://saves.mbi.ucla.edu/). The model was manually check with WinCoot V0.8.9. For the PtdIns3K complex structure, by comparison we used the structure of the latest resolved PI3KC3-C1 complex (PDB ID: 8SOR). The full-length protein binding modes of APPL2 and PtdIns3K complex were studied by using GRAMM-X, which is guided by the experimental details available for their interactions and functions. The protein-protein binding modes were studied and analyzed by using PDBePISA server. The results were analyzed by using PyMOL 1.8.6 to characterize critical amino acids in the protein-protein/peptide interaction interface. (red: APPL2, green: PIK3R4, yellow: PIK3C3, blue: ATG14, pink: BECLIN-1.)

Supplementary Table S1 Detailed list of materials used in this study

ANTIBODIES	SOURCE	IDENTIFIER
APPL2,1:1000	Proteintech	Cat#14294-1-AP
APPL2,1:200	ABclonal	Cat#A14590
CDKN1A,1:500	Proteintech	Cat#10355-1-AP
CDKN2A,1：1000	ABclonal	Cat#A11058
GFP Rabbit Monoclonal Antibody,1:1000	Beyotime	Cat#AF1483
β-actin,1:1000	Proteintech	Cat#20536-1-AP
GAPDH,1:4000	Proteintech	Cat#10494-1-AP
IRDye 800CW Goat anti-Rabbit IgG (H+L),1:10000	LI-COR	Cat#926-32211
HRP was used to label goat anti-rabbit secondary antibody,1:200	Servicebio	Cat#GB23303

REAGENTS	SOURCE	IDENTIFIER
Gelatin from bovine skin	SIGMA	Cat#G9391
Collagenase, Type 1	Diamond	Cat#A004194
Endothelial Cell Medium	ScienCell	Cat#1001
DMEM High Glucose	VivaCell BIOSCIENCES	Cat#C3110-0500
Opti-MEMTM	Gibco	Cat#31985-070
Fetal bovine serum (FBS)	EVERY GREEN	Cat#11011-8611
Penicillin-Streptomycin Liquid	Solarbio	Cat#P1400
Polybrene (Hexadimethrine Bromide)	Beyotime	Cat#C0351
Polyethylenimine	Polysciences	Cat#23966-1
Lipofectamine™ 2000	Thermo	Cat#11668019
Puromycin Dihydrochloride	Beyotime	Cat#ST551
BSA	Meilunbio	Cat#9048-46-8
T4 DNA Ligase	TaKaLa	Cat#2011A
2 × Phanta Max Master Mix	Vazyme	Cat#P515-02
EndoFree Plamisid Midi Kit	CWBIO	Cat#CW2105S
SanPrep Column PCR Product Purification Kit	Sangon Biotech	Cat#B518141-0100
Gel Extraction Kit	CWBIO	Cat#CW2302M
RaPure Total RNA Kit	Magen	Cat#R4011-02
NGzol RNA Uptake Kit	HLINGENE	Cat#NG304S
HiScript II Q RT SuperMix for qPCR	Vazyme	Cat#R223
ChamQ Universal SYBR qPCR Master Mix	Vazyme	Cat#Q711-02
Cholesterol	Diamond	Cat#A100433

Chloroquine diphosphate salt	SIGMA	Cat#C6628
Rapamycin(Sirolimus)	APExBIO	Cat#A8167
TritonX-100	Solarbio	Cat#T8200
Senescence β-Galactosidase Staining Kit	Beyotime	Cat#C0602
tert-ButyI hydroperoxide solution (TBHP)	MACKLIN	Cat#B802372
Modified Oil Red O Staining Kit	Beyotime	Cat#C0158S
Reactive Oxygen Species Assay Kit	Beyotime	Cat#S0033S
**CELLS**	**SOURCE**	**IDENTIFIER**
HEK293T cells	ATCC	Cat#CBP60439
HUVECs	In our lab	N/A
DH-5α	Shanghai Weidi Biotechnology	Cat#DL1001
**ANIMALS**	**SOURCE**	**IDENTIFIER**
Mouse: WT C57BL6/J	Laboratory Animal Center, Zhengzhou University	No.
Caenorhabditis elegans:N2（WT）	Professor Ding Chunbang from Sichuan Agricultural University，China	N/A
Caenorhabditis elegans:DA2123:adls2122(lgg-1p::gfp::lgg-1)	Dr Zhang Hong from Institute of Biophysics，Chinese Academy of Sciences，China	N/A
**PLASMIDS**	**SOURCE**	**IDENTIFIER**
pLKO.1-TRC Cloning Vector	Addgene	Cat#10878
L4440	Addgene	Cat#1654
**SOFTWARES**	**SOURCE**	
Graphpad Prism for statistical analysis	GraphPad	
ImageJ	NIH	
Image Studio	Alias	
Snapgene Viewer	GSL Biotech LLC	
DNAman	LynnonBiosoft	
Odyssey Application Software	Li-cor Biosciences	
Image-Pro Plus software	Media Cybernetics	
Zen	ZEISS	
Molecular Evolutionary Genetics Analysis	Center for Evolutionary Medicine and Informatics	
GeneDoc	Karl Nicholas	

Supplementary Table S2 RT-PCR primer sequences

**Gene**	**Forward primer**	**Reverse primer**
APPL2(M)	GCTAAGTCTCAAGTCCCCGAG	TGGTGTAATCTGTGAGGGTGC
GAPDH(M)	AGGGCCCACTGAAGGGCATCTT	ATTGAGAGCAATGCCAGCCCCG
T04C9.1(C)	AGAAGAGACTGTAGCTGCGA	CCGACTCCGAAAGATCGTGT
let-363(C)	AGTGAGCTGAAAGACGAGCC	TCAGCTAAGCACACGATGAGA
UNC-51(C)	AAAAGGGCATCGTACATCGT	ATTTTGGGTGCGGGAGTT
EPG-1(C)	ATCCAGCAAATGGAACCAAG	TGGAGTTGATTTTGGAGAATTG
EPG-3(C)	CGAGTGTCAGAGCCTGGATT	CTTTTTGTTGAGGGGCATTG
EPG-9(C)	GAAGTCAGTTGTGAGCAGATTGA	TGGCTTCTTCCACTTCACATT
VPS-34(C)	AACCCTGTCAGAAGGTTGAATC	TGACGAGCAAGTTGAGAGGA
VPS-15(C)	CGATCGATTGAGCACGAG	TGAAGAGCAGGAAGATGTACCA
ATG-16.1(C)	CAGAAGTTGCTTTAGAAGAAAAACG	TTTTGTGTCCTTGTCGGTGA
ATG-7(C)	TCTTGCTATTTCTGCAGTGATGT	GTTCCTGGTCGTGCAACAG
ATG-2(C)	CTCAACCACATGGTGTCGTC	CATCGGTATGGAAAGTAACACCA
ATG-9(C)	GGTCTTCACGATGAGAGTATTATCC	TGCATGTTGAAGCTTGACG
EPG-5(C)	GCGCCAGGATTAGTAGTCAAG	CCAATTGAGGCCAATGAGTT
ACTIN-1(C)	CAAGAGAGGTATCCTTACCCTCAAG	GTCCGGAAGCGTAGAGGGAG
